# The genome sequence of the Soprano Pipistrelle, *Pipistrellus pygmaeus *(Leach, 1825)

**DOI:** 10.12688/wellcomeopenres.19895.1

**Published:** 2023-08-21

**Authors:** Manuel Ruedi, Sonja C. Vernes, Emma C. Teeling, Meike Mai, Wellcome Sanger Institute Tree of Life programme, Wellcome Sanger Institute Scientific Operations: DNA Pipelines collective, Tree of Life Core Informatics collective, Darwin Tree of Life Consortium

**Affiliations:** 1 Muséum d'histoire naturelle de Genève, Geneva, Switzerland; 2 School of Biology, University of St Andrews, St Andrews, Scotland, UK; 3 Neurogenetics of Vocal Communication Group, Max Planck Institute for Psycholinguistics, Nijmegen, Gelderland, The Netherlands; 4 School of Biology and Environmental Science, University College Dublin, Dublin, Leinster, Ireland; 5 Wellcome Sanger Institute, Hinxton, England, UK

**Keywords:** Pipistrellus pygmaeus, Soprano Pipistrelle, genome sequence, chromosomal, Chiroptera

## Abstract

We present a genome assembly from an individual male *Pipistrellus pygmaeus* (the Soprano Pipistrelle; Chordata; Mammalia; Chiroptera; Vespertilionidae). The genome sequence is 1,895.1 megabases in span. Most of the assembly is scaffolded into 23 chromosomal pseudomolecules, including the X and Y sex chromosomes. The mitochondrial genome has also been assembled and is 17.18 kilobases in length.

## Species taxonomy

Eukaryota; Metazoa; Eumetazoa; Bilateria; Deuterostomia; Chordata; Craniata; Vertebrata; Gnathostomata; Teleostomi; Euteleostomi; Sarcopterygii; Dipnotetrapodomorpha; Tetrapoda; Amniota; Mammalia; Theria; Eutheria; Boreoeutheria; Laurasiatheria; Chiroptera; Yangochiroptera; Vespertilionoidea; Vespertilionidae; *Pipistrellus* (Leach, 1825) (NCBI:txid246814; subordinal taxonomy updated per Teeling *et al.*, 2005).

## Background

**Figure 1.**
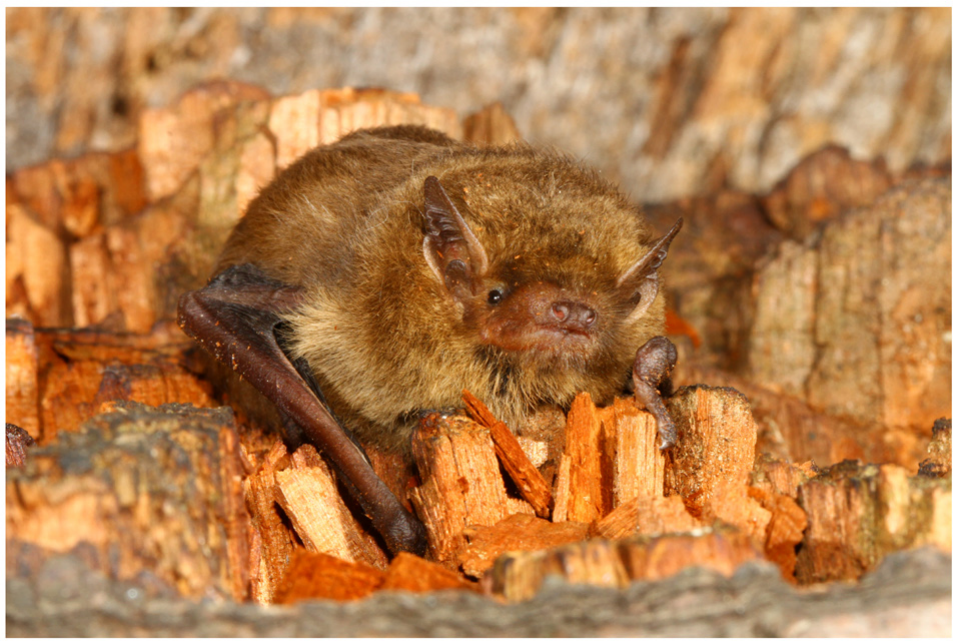
Photograph of a *Pipistrellus pygmaeus* by Manuel Ruedi.

The Soprano pipistrelle ( *Pipistrellus pygmaeus*) ( Figure 1) is a common bat found throughout most of Europe where it lives in urbanised, as well as in a variety of riparian habitats, often near lakes or large rivers ( Davidson-Watts *et al.*, 2006). With an average adult weight of 3–4 g, it is the smallest European species of bat. During the summer, breeding females may aggregate into large colonies of up to a thousand individuals ( Oakeley & Jones, 1998). Nursery colonies are typically installed under roofs, attics, behind shutters or in any kind of cracks in buildings. Because of this anthropophilic behaviour, the Soprano Pipistrelle is often abundant and does not suffer from any major threats in Europe. It is classified as Least Concern (LC) under IUCN criteria.

Populations in parts of their range appear to be migratory (e.g. in the Balkans) whereas others, such as in Western Europe, do not seem to engage in significant migrations ( Bryja *et al.*, 2008). Interestingly, this bat has been known to science only very recently (its species status was recognised in 1997 ( Barratt *et al.*, 1997)) owing to its species typical echolocation calls, which are produced at 55 kHz and distinguish it from those of the Common Pipistrelle ( *Pipistrellus pipistrellus*), calling at 45 kHz ( Barlow & Jones, 1997). Morphologically, however, both species are extremely similar, and may live in sympatry in many parts of Europe ( Davidson-Watts *et al.*, 2006).

The genome of this species has not been characterised previously, but extensive phylogeographic studies based on mitochondrial ( Hulva *et al.*, 2004) or few nuclear loci ( Bryja *et al.*, 2008) have been published in order to understand its molecular distinction from the sibling species *P. pipistrellus*. This new, complete genome of a *P. pygmaeus* will therefore provide an unprecedented resolution into the evolution of these two cryptic species, given that a comparative genome for the common pipistrelle is already available.

We present a chromosomally complete genome sequence for *Pipistrellus pygmaeus*, based on one male specimen from Geneva, Switzerland, as part of the Darwin Tree of Life Project and Bat1K Project ( Teeling *et al.*, 2018). This project is a collaborative effort to sequence all named eukaryotic species in the Atlantic Archipelago of Britain and Ireland.

## Genome sequence report

The genome was sequenced from one male *Pipistrellus pygmaeus* collected from Geneva, Switzerland (46.19, 6.17). A total of 36-fold coverage in Pacific Biosciences single-molecule HiFi long reads was generated. Primary assembly contigs were scaffolded with chromosome conformation Hi-C data. Manual assembly curation corrected 16 missing joins or misjoins and removed 7 haplotypic duplications, reducing the assembly length by 0.43% and the scaffold number by 6.18%, and increasing the scaffold N50 by 0.5%.

**Figure 2.**
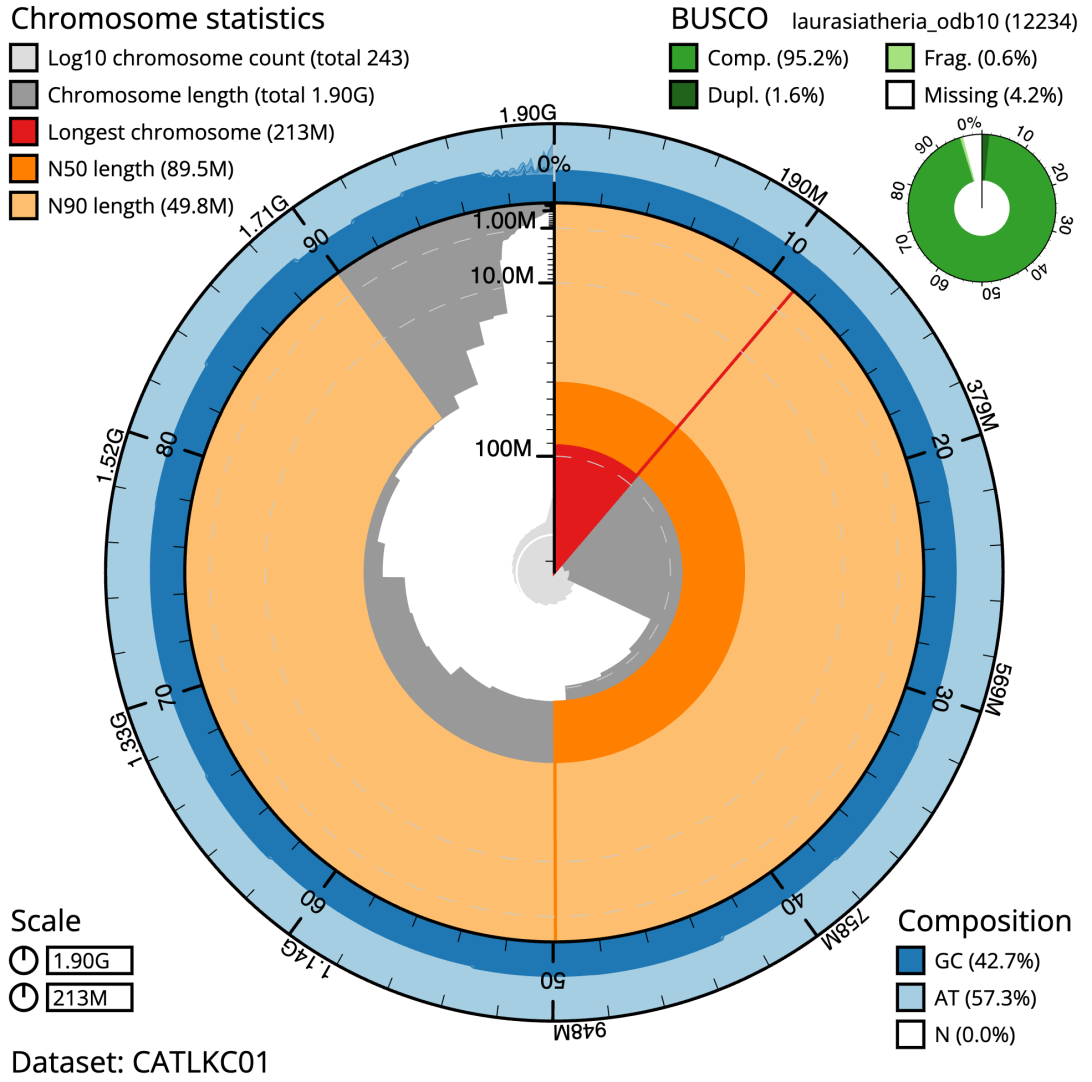
Genome assembly of *Pipistrellus pygmaeus*, mPipPyg2.1: metrics. The BlobToolKit Snailplot shows N50 metrics and BUSCO gene completeness. The main plot is divided into 1,000 size-ordered bins around the circumference with each bin representing 0.1% of the 1,895,125,685 bp assembly. The distribution of scaffold lengths is shown in dark grey with the plot radius scaled to the longest scaffold present in the assembly (212,679,785 bp, shown in red). Orange and pale-orange arcs show the N50 and N90 scaffold lengths (89,507,134 and 49,778,105 bp), respectively. The pale grey spiral shows the cumulative scaffold count on a log scale with white scale lines showing successive orders of magnitude. The blue and pale-blue area around the outside of the plot shows the distribution of GC, AT and N percentages in the same bins as the inner plot. A summary of complete, fragmented, duplicated and missing BUSCO genes in the laurasiatheria_odb10 set is shown in the top right. An interactive version of this figure is available at https://blobtoolkit.genomehubs.org/view/mPipPyg2.1/dataset/CATLKC01/snail.

The final assembly has a total length of 1,895.1 Mb in 242 sequence scaffolds with a scaffold N50 of 89.5 Mb ( Table 1). Most (97.34%) of the assembly sequence was assigned to 23 chromosomal-level scaffolds, representing 21 autosomes and the X and Y sex chromosomes. The sex chromosomes were assigned by coverage statistics. Chromosome-scale scaffolds confirmed by the Hi-C data are named in order of size ( Figure 2– Figure 5; Table 2). While not fully phased, the assembly deposited is of one haplotype. Contigs corresponding to the second haplotype have also been deposited. The mitochondrial genome was also assembled and can be found as a contig within the multifasta file of the genome submission.

**Table 1.** Genome data for *Pipistrellus pygmaeus*, mPipPyg2.1.

Project accession data		
Assembly identifier	mPipPyg2.1	
Species	*Pipistrellus pygmaeus*	
Specimen	mPipPyg2	
NCBI taxonomy ID	246814	
BioProject	PRJEB61049	
BioSample ID	SAMEA9921456	
Isolate information	mPipPyg2, male: heart and muscle (DNA sequencing and Hi-C scaffolding) mPipPyg3, female: liver (RNA sequencing)	
Assembly metrics *		*Benchmark*
Consensus quality (QV)	61	*≥ 50*
*k*-mer completeness	100%	*≥ 95%*
BUSCO **	C:95.2%[S:93.6%,D:1.6%], F:0.6%,M:4.2%,n:12,234	*C ≥ 95%*
Percentage of assembly mapped to chromosomes	97.34%	*≥ 95%*
Sex chromosomes	X and Y	*localised* *homologous* *pairs*
Organelles	Mitochondrial genome assembled	*complete single* *alleles*
Raw data accessions		
PacificBiosciences SEQUEL IIe	ERR11180455, ERR11180456, ERR11180457	
Hi-C Illumina	ERR11182530	
PolyA RNA-Seq Illumina	ERR11641135	
Genome assembly		
Assembly accession	GCA_949987585.1	
*Accession of alternate* *haplotype*	GCA_949987765.1	
Span (Mb)	1,895.1	
Number of contigs	404	
Contig N50 length (Mb)	54.0	
Number of scaffolds	242	
Scaffold N50 length (Mb)	89.5	
Longest scaffold (Mb)	212.7	

* Assembly metric benchmarks are adapted from column VGP-2020 of “Table 1: Proposed standards and metrics for defining genome assembly quality” from Rhie *et al.* (2021). ** BUSCO scores based on the laurasiatheria_odb10 BUSCO set using v5.3.2. C = complete [S = single copy, D = duplicated], F = fragmented, M = missing, n = number of orthologues in comparison. A full set of BUSCO scores is available at https://blobtoolkit.genomehubs.org/view/mPipPyg2.1/dataset/CATLKC01/busco.

**Figure 3.**
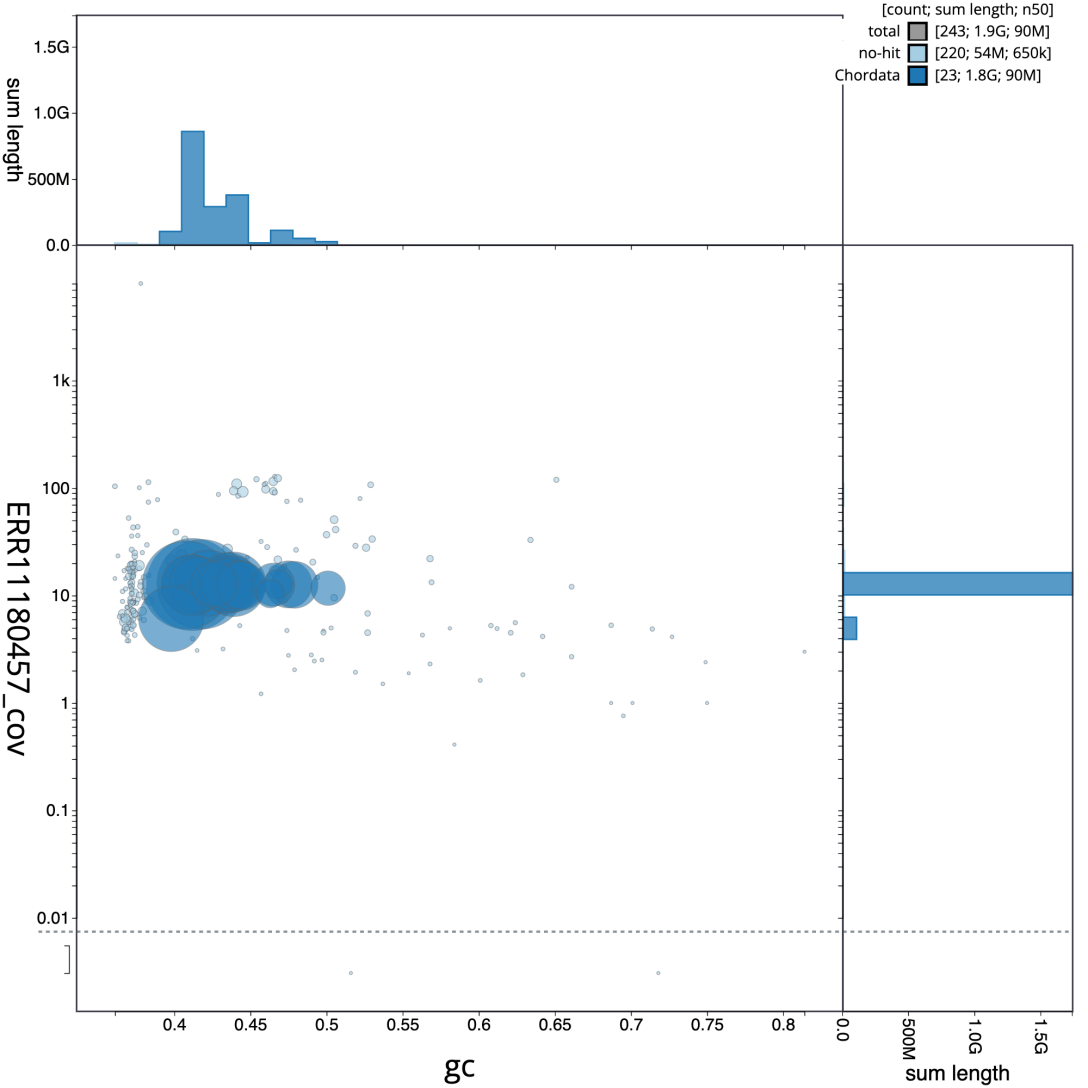
Genome assembly of *Pipistrellus pygmaeus*, mPipPyg2.1: BlobToolKit GC-coverage plot. Scaffolds are coloured by phylum. Circles are sized in proportion to scaffold length. Histograms show the distribution of scaffold length sum along each axis. An interactive version of this figure is available at https://blobtoolkit.genomehubs.org/view/mPipPyg2.1/dataset/CATLKC01/blob.

**Figure 4.**
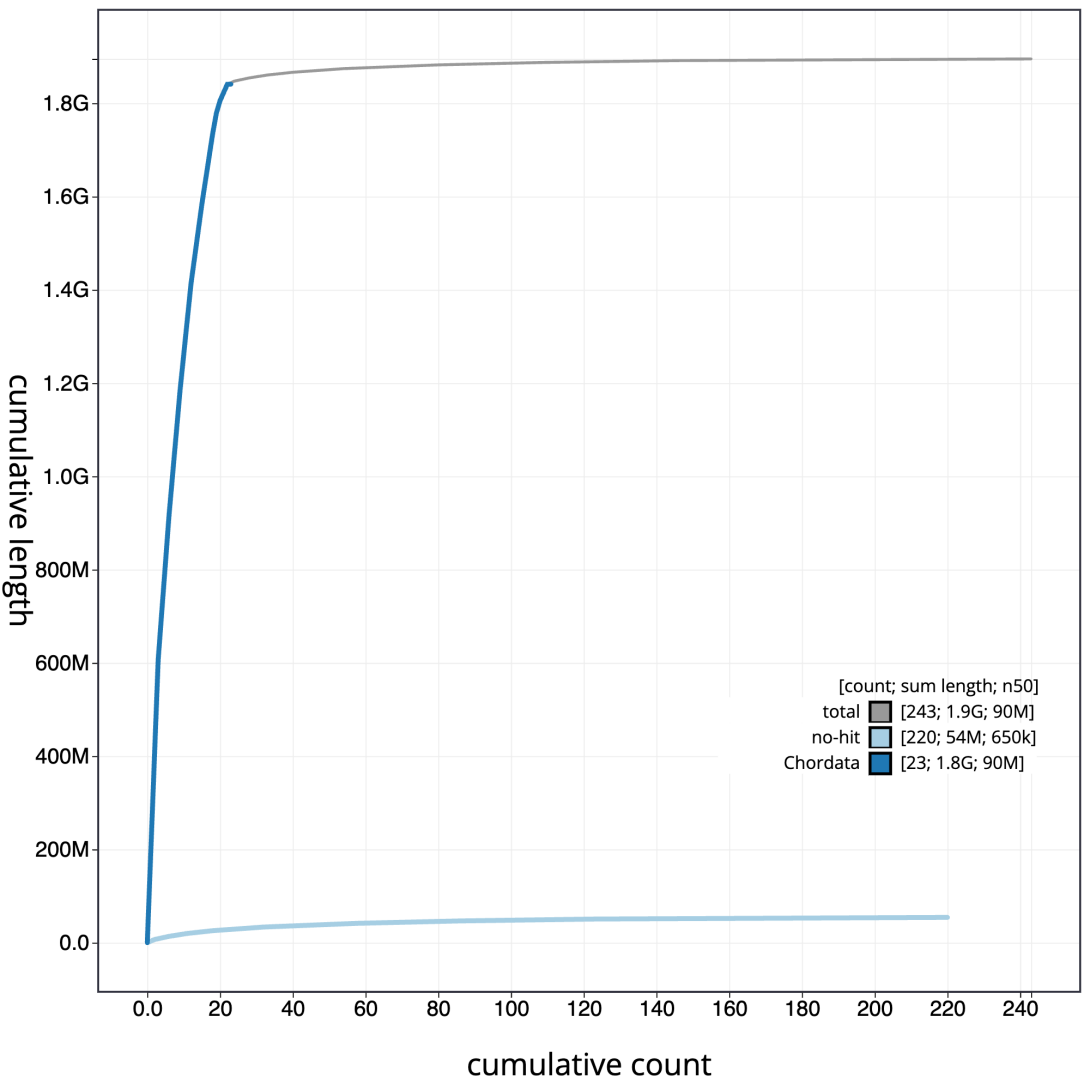
Genome assembly of *Pipistrellus pygmaeus*, mPipPyg2.1: BlobToolKit cumulative sequence plot. The grey line shows cumulative length for all scaffolds. Coloured lines show cumulative lengths of scaffolds assigned to each phylum using the buscogenes taxrule. An interactive version of this figure is available at https://blobtoolkit.genomehubs.org/view/mPipPyg2.1/dataset/CATLKC01/cumulative.

**Figure 5.**
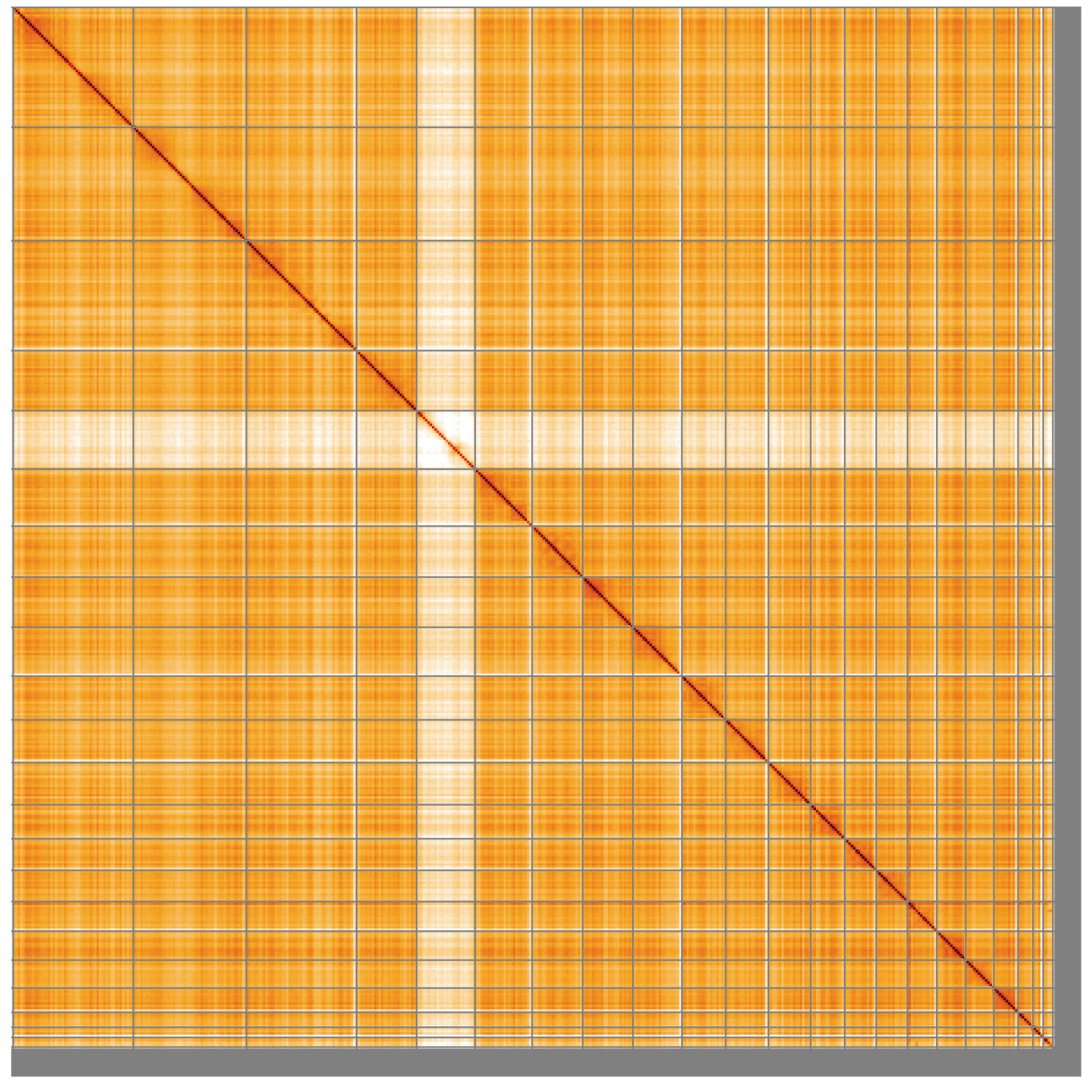
Genome assembly of *Pipistrellus pygmaeus*, mPipPyg2.1: Hi-C contact map of the mPipPyg2.1 assembly, visualised using HiGlass. Chromosomes are shown in order of size from left to right and top to bottom. An interactive version of this figure may be viewed at https://genome-note-higlass.tol.sanger.ac.uk/l/?d=CzJBxflARPCHULHU6AtV-A.

**Table 2.** Chromosomal pseudomolecules in the genome assembly of *Pipistrellus pygmaeus*, mPipPyg2.

INSDC accession	Chromosome	Length (Mb)	GC%
OX465302.1	1	212.68	41.0
OX465303.1	2	200.21	42.0
OX465304.1	3	194.78	41.0
OX465305.1	4	106.54	42.0
OX465307.1	5	100.97	44.0
OX465308.1	6	89.51	43.5
OX465309.1	7	89.07	41.0
OX465310.1	8	86.55	41.0
OX465311.1	9	77.44	44.0
OX465312.1	10	75.91	41.5
OX465313.1	11	74.62	43.5
OX465314.1	12	60.36	43.5
OX465315.1	13	55.71	44.5
OX465316.1	14	55.55	44.5
OX465317.1	15	51.81	47.5
OX465318.1	16	51.16	48.0
OX465319.1	17	49.78	42.5
OX465320.1	18	42.76	46.5
OX465321.1	19	26.46	50.0
OX465322.1	20	18.02	47.0
OX465323.1	21	17.27	46.5
OX465306.1	X	103.57	40.0
OX465324.1	Y	4.17	42.5
OX465325.1	MT	0.02	38.0

The estimated Quality Value (QV) of the final assembly is 61 with *k*-mer completeness of 100%, and the assembly has a BUSCO v5.3.2 completeness of 95.2% (single = 93.6%, duplicated = 1.6%), using the laurasiatheria_odb10 reference set ( *n* = 12,234).

Metadata for specimens, spectral estimates, sequencing runs, contaminants and pre-curation assembly statistics can be found at https://links.tol.sanger.ac.uk/species/246814.

## Methods

### Sample acquisition and nucleic acid extraction

The specimen used for DNA sequencing was a male *P. pygmaeus* (specimen ID SAN0001697, ToLID mPipPyg2), which was collected in Geneva, Switzerland (latitude 46.19, longitude 6.17) on 2021-06-17. The specimen used for RNA sequencing was a female *P. pygmaeus* (ToLID mPipPyg3), collected in Geneva, Switzerland (latitude 46.19, 6.12) on 2016-10-03. Both specimens were injured, euthanised bats, from an urban city habitat. The specimens were collected and identified by Manuel Ruedi (Natural History Museum Geneva). The whole specimens are now preserved in 80% ethanol, at room temperature with associated catalogue number MHNG-MAMO-3010.006 and 3002.006, respectively.

DNA was extracted at the Tree of Life laboratory, Wellcome Sanger Institute (WSI). The mPipPyg2 sample was weighed and dissected on dry ice with tissue set aside for Hi-C sequencing. Muscle and heart tissue was cryogenically disrupted to a fine powder using a Covaris cryoPREP Automated Dry Pulveriser, receiving multiple impacts. High molecular weight (HMW) DNA was extracted using the Qiagen MagAttract HMW DNA extraction kit. HMW DNA was sheared into an average fragment size of 12–20 kb in a Megaruptor 3 system with speed setting 30. Sheared DNA was purified by solid-phase reversible immobilisation using AMPure PB beads with a 1.8X ratio of beads to sample to remove the shorter fragments and concentrate the DNA sample. The concentration of the sheared and purified DNA was assessed using a Nanodrop spectrophotometer and Qubit Fluorometer and Qubit dsDNA High Sensitivity Assay kit. Fragment size distribution was evaluated by running the sample on the FemtoPulse system.

RNA was extracted from liver tissue of mPipPyg3 in the Tree of Life Laboratory at the WSI using TRIzol, according to the manufacturer’s instructions. RNA was then eluted in 50 μl RNAse-free water and its concentration assessed using a Nanodrop spectrophotometer and Qubit Fluorometer using the Qubit RNA Broad-Range (BR) Assay kit. Analysis of the integrity of the RNA was done using Agilent RNA 6000 Pico Kit and Eukaryotic Total RNA assay.

### Sequencing

Pacific Biosciences HiFi circular consensus DNA sequencing libraries were constructed according to the manufacturers’ instructions. Poly(A) RNA-Seq libraries were constructed using the NEB Ultra II RNA Library Prep kit. DNA and RNA sequencing was performed by the Scientific Operations core at the WSI on Pacific Biosciences SEQUEL II (HiFi) and Illumina NovaSeq 6000 (RNA-Seq) instruments. Hi-C data were also generated from muscle tissue of mPipPyg2 using the Arima2 kit and sequenced on the Illumina NovaSeq 6000 instrument.

### Genome assembly, curation and evaluation

Assembly was carried out with Hifiasm ( Cheng *et al.*, 2021) and haplotypic duplication was identified and removed with purge_dups ( Guan *et al.*, 2020). One round of polishing was performed by aligning 10X Genomics read data to the assembly with Long Ranger ALIGN, calling variants with FreeBayes ( Garrison & Marth, 2012). The assembly was then scaffolded with Hi-C data ( Rao *et al.*, 2014) using YaHS ( Zhou *et al.*, 2023) [OR SALSA2 ( Ghurye *et al.*, 2019)]. The assembly was checked for contamination and corrected using the gEVAL system ( Chow *et al.*, 2016) as described previously ( Howe *et al.*, 2021). Manual curation was performed using gEVAL, HiGlass ( Kerpedjiev *et al.*, 2018) and Pretext ( Harry, 2022). The mitochondrial genome was assembled using MitoHiFi ( Uliano-Silva *et al.*, 2023), which runs MitoFinder ( Allio *et al.*, 2020) or MITOS ( Bernt *et al.*, 2013) and uses these annotations to select the final mitochondrial contig and to ensure the general quality of the sequence.

A Hi-C map for the final assembly was produced using bwa-mem2 ( Vasimuddin *et al.*, 2019) in the Cooler file format ( Abdennur & Mirny, 2020). To assess the assembly metrics, the *k*-mer completeness and QV consensus quality values were calculated in Merqury ( Rhie *et al.*, 2020). This work was done using Nextflow ( Di Tommaso *et al.*, 2017) DSL2 pipelines “sanger-tol/readmapping” ( Surana *et al.*, 2023a) and “sanger-tol/genomenote” ( Surana *et al.*, 2023b). The genome was analysed within the BlobToolKit environment ( Challis *et al.*, 2020) and BUSCO scores ( Manni *et al.*, 2021; Simão *et al.*, 2015) were calculated.

Table 3 contains a list of relevant software tool versions and sources.

**Table 3.** Software tools: versions and sources.

Software tool	Version	Source
BlobToolKit	4.1.7	https://github.com/blobtoolkit/blobtoolkit
BUSCO	5.3.2	https://gitlab.com/ezlab/busco
Hifiasm	0.16.1-r375	https://github.com/chhylp123/hifiasm
HiGlass	1.11.6	https://github.com/higlass/higlass
Merqury	MerquryFK	https://github.com/thegenemyers/MERQURY.FK
MitoHiFi	3	https://github.com/marcelauliano/MitoHiFi
PretextView	0.2	https://github.com/wtsi-hpag/PretextView
purge_dups	1.2.3	https://github.com/dfguan/purge_dups
sanger-tol/ genomenote	v1.0	https://github.com/sanger-tol/genomenote
sanger-tol/ readmapping	1.1.0	https://github.com/sanger-tol/readmapping/tree/1.1.0
YaHS	1.2a.2	https://github.com/c-zhou/yahs

### Wellcome Sanger Institute – Legal and Governance

The materials that have contributed to this genome note have been supplied by a Tree of Life collaborator. The Wellcome Sanger Institute employs a process whereby due diligence is carried out proportionate to the nature of the materials themselves, and the circumstances under which they have been/are to be collected and provided for use. The purpose of this is to address and mitigate any potential legal and/or ethical implications of receipt and use of the materials as part of the research project, and to ensure that in doing so we align with best practice wherever possible. The overarching areas of consideration are:

•   Ethical review of provenance and sourcing of the material

•   Legality of collection, transfer and use (national and international)

Each transfer of samples is undertaken according to a Research Collaboration Agreement or Material Transfer Agreement entered into by the Tree of Life collaborator, Genome Research Limited (operating as the Wellcome Sanger Institute) and in some circumstances other Tree of Life collaborators.

## Data Availability

European Nucleotide Archive: *Pipistrellus pygmaeus* (soprano pipistrelle). Accession number PRJEB61049; https://identifiers.org/ena.embl/PRJEB61049. ( Wellcome Sanger Institute, 2023) The genome sequence is released openly for reuse. The *Pipistrellus pygmaeus* genome sequencing initiative is part of the Darwin Tree of Life (DToL) project. All raw sequence data and the assembly have been deposited in INSDC databases. The genome will be annotated using available RNA-Seq data and presented through the Ensembl pipeline at the European Bioinformatics Institute. Raw data and assembly accession identifiers are reported in Table 1.

## Author information

Members of the Wellcome Sanger Institute Tree of Life programme are listed here: https://doi.org/10.5281/zenodo.4783585.

Members of Wellcome Sanger Institute Scientific Operations: DNA Pipelines collective are listed here: https://doi.org/10.5281/zenodo.4790455.

Members of the Tree of Life Core Informatics collective are listed here: https://doi.org/10.5281/zenodo.5013541.

Members of the Darwin Tree of Life Consortium are listed here: https://doi.org/10.5281/zenodo.4783558.

## References

[ref-1] Abdennur N Mirny LA : Cooler: Scalable storage for Hi-C data and other genomically labeled arrays. *Bioinformatics.* 2020;36(1):311–316. 10.1093/bioinformatics/btz540 31290943 PMC8205516

[ref-2] Allio R Schomaker-Bastos A Romiguier J : MitoFinder: Efficient automated large‐scale extraction of mitogenomic data in target enrichment phylogenomics. *Mol Ecol Resour.* 2020;20(4):892–905. 10.1111/1755-0998.13160 32243090 PMC7497042

[ref-3] Barlow KE Jones G : Differences in songflight calls and social calls between two phonic types of the vespertilionid bat *Pipistrellus pipistrellus.* *J Zool.* 1997;241(2):315–324. 10.1111/j.1469-7998.1997.tb01962.x

[ref-4] Barratt EM Deaville R Burland TM : DNA answers the call of pipistrelle bat species. *Nature.* 1997;387(6629):138–139. 10.1038/387138b0 9144281

[ref-5] Bernt M Donath A Jühling F : MITOS: Improved *de novo* metazoan mitochondrial genome annotation. *Mol Phylogenet Evol.* 2013;69(2):313–319. 10.1016/j.ympev.2012.08.023 22982435

[ref-6] Bryja J Kaňuch P Fornůsková A : Low population genetic structuring of two cryptic bat species suggests their migratory behaviour in continental Europe. *Biol J Linn Soc Lond.* 2008;96(1):103–114. 10.1111/j.1095-8312.2008.01093.x

[ref-7] Challis R Richards E Rajan J : BlobToolKit - interactive quality assessment of genome assemblies. *G3 (Bethesda).* 2020;10(4):1361–1374. 10.1534/g3.119.400908 32071071 PMC7144090

[ref-8] Cheng H Concepcion GT Feng X : Haplotype-resolved *de novo* assembly using phased assembly graphs with hifiasm. *Nat Methods.* 2021;18(2):170–175. 10.1038/s41592-020-01056-5 33526886 PMC7961889

[ref-9] Chow W Brugger K Caccamo M : gEVAL — a web-based browser for evaluating genome assemblies. *Bioinformatics.* 2016;32(16):2508–2510. 10.1093/bioinformatics/btw159 27153597 PMC4978925

[ref-10] Davidson-Watts I Walls S Jones G : Differential habitat selection by *Pipistrellus pipistrellus* and *Pipistrellus pygmaeus* identifies distinct conservation needs for cryptic species of echolocating bats. *Biol Conserv.* 2006;133(1):118–127. 10.1016/j.biocon.2006.05.027

[ref-11] Di Tommaso P Chatzou M Floden EW : Nextflow enables reproducible computational workflows. *Nat Biotechnol.* 2017;35(4):316–319. 10.1038/nbt.3820 28398311

[ref-12] Garrison E Marth G : Haplotype-based variant detection from short-read sequencing. 2012; Accessed 26 July 2023 . Reference Source

[ref-13] Ghurye J Rhie A Walenz BP : Integrating Hi-C links with assembly graphs for chromosome-scale assembly. *PLoS Comput Biol.* 2019;15(8): e1007273. 10.1371/journal.pcbi.1007273 31433799 PMC6719893

[ref-14] Guan D McCarthy SA Wood J : Identifying and removing haplotypic duplication in primary genome assemblies. *Bioinformatics.* 2020;36(9):2896–2898. 10.1093/bioinformatics/btaa025 31971576 PMC7203741

[ref-15] Harry E : PretextView(Paired REad TEXTure Viewer): A desktop application for viewing pretext contact maps. 2022; Accessed 19 October 2022. Reference Source

[ref-16] Howe K Chow W Collins J : Significantly improving the quality of genome assemblies through curation. *GigaScience.* Oxford University Press,2021;10(1): giaa153. 10.1093/gigascience/giaa153 33420778 PMC7794651

[ref-17] Hulva P Horáček I Strelkov PP : Molecular architecture of *Pipistrellus pipistrellus*/ *Pipistrellus pygmaeus* complex (Chiroptera: Vespertilionidae): further cryptic species and Mediterranean origin of the divergence. *Mol Phylogenet Evol.* 2004;32(3):1023–1035. 10.1016/j.ympev.2004.04.007 15288073

[ref-18] Kerpedjiev P Abdennur N Lekschas F : HiGlass: web-based visual exploration and analysis of genome interaction maps. *Genome Biol. * 2018;19(1): 125. 10.1186/s13059-018-1486-1 30143029 PMC6109259

[ref-19] Manni M Berkeley MR Seppey M : BUSCO update: Novel and streamlined workflows along with broader and deeper phylogenetic coverage for scoring of eukaryotic, prokaryotic, and viral genomes. *Mol Biol Evol.* 2021;38(10):4647–4654. 10.1093/molbev/msab199 34320186 PMC8476166

[ref-20] Oakeley SF Jones G : Habitat around maternity roosts of the 55 kHz phonic type of pipistrelle bats ( *Pipistrellus pipistrellus*). *J Zool.* 1998;245(2):222–228. 10.1111/j.1469-7998.1998.tb00094.x

[ref-21] Rao SSP Huntley MH Durand NC : A 3D map of the human genome at kilobase resolution reveals principles of chromatin looping. *Cell.* 2014;159(7):1665–1680. 10.1016/j.cell.2014.11.021 25497547 PMC5635824

[ref-22] Rhie A McCarthy SA Fedrigo O : Towards complete and error-free genome assemblies of all vertebrate species. *Nature.* 2021;592(7856):737–746. 10.1038/s41586-021-03451-0 33911273 PMC8081667

[ref-23] Rhie A Walenz BP Koren S : Merqury: Reference-free quality, completeness, and phasing assessment for genome assemblies. *Genome Biol.* 2020;21(1): 245. 10.1186/s13059-020-02134-9 32928274 PMC7488777

[ref-24] Simão FA Waterhouse RM Ioannidis P : BUSCO: assessing genome assembly and annotation completeness with single-copy orthologs. *Bioinformatics.* 2015;31(19):3210–3212. 10.1093/bioinformatics/btv351 26059717

[ref-25] Surana P Muffato M Qi G : sanger-tol/readmapping: sanger-tol/readmapping v1.1.0 - Hebridean Black(1.1.0). *Zenodo.* 2023a; Accessed 21 July 2023. 10.5281/zenodo.7755665

[ref-26] Surana P Muffato M Sadasivan Baby C : sanger-tol/genomenote (v1.0.dev). *Zenodo.* 2023b; Accessed 21 July 2023. Reference Source

[ref-27] Teeling EC Springer MS Madsen O : A Molecular Phylogeny for Bats Illuminates Biogeography and the Fossil Record. *Science.* 2005;307(5709):580–584. 10.1126/science.1105113 15681385

[ref-28] Teeling EC Vernes SC Dávalos LM : Bat Biology, Genomes, and the Bat1K Project: To Generate Chromosome-Level Genomes for All Living Bat Species. *Annu Rev Anim Biosci.* 2018;6:23–46. 10.1146/annurev-animal-022516-022811 29166127

[ref-29] Uliano-Silva M Ferreira JGRN Krasheninnikova K : MitoHiFi: a python pipeline for mitochondrial genome assembly from PacBio high fidelity reads. *BMC Bioinformatics.* 2023;24(1): 288. 10.1186/s12859-023-05385-y 37464285 PMC10354987

[ref-30] Vasimuddin Md Misra S Li H : Efficient Architecture-Aware Acceleration of BWA-MEM for Multicore Systems. *2019 IEEE International Parallel and Distributed Processing Symposium(IPDPS).*IEEE,2019;314–324. 10.1109/IPDPS.2019.00041

[ref-50] Wellcome Sanger Institute: The genome sequence of the Soprano Pipistrelle, *Pipistrellus pygmaeus* (Leach, 1825). *European Nucleotide Archive.* [dataset], accession number PRJEB61049,2023 10.12688/wellcomeopenres.19895.1 PMC11101169 38764969

[ref-31] Zhou C McCarthy SA Durbin R : YaHS: yet another Hi-C scaffolding tool. *Bioinformatics.* 2023;39(1): btac808. 10.1093/bioinformatics/btac808 36525368 PMC9848053

