# Supplementary material for: The genome sequence of the Soprano Pipistrelle, Pipistrellus pygmaeus (Leach, 1825)
Source: Wellcome Open Res. 2023 Aug 21;8:360. [Version 1] doi: 10.12688/wellcomeopenres.19895.1 (PMC11101169; doi:10.12688/wellcomeopenres.19895.1)
Supplement: Peer review history

# Reviewer report: Reviewer response for version 1

**DOI:** 10.21956/wellcomeopenres.22030.r81224

**Published:** 2024-05-16

**Authors:** Balaji Chattopadhyay

**Affiliations:** 1 Ashoka University, Haryana, India

Reudi et al. has presented a high quality assembly the Soprano Pipistrelle, *Pipistrellus pygmaeus. *The authors have assembled the genome into mitochondrial genome and chromosomal pseudomolecules including sex chromosomes.

 Overall the study serves as an important guide for researchers and students who are interested in high quality genome sequencing and assembly of natural populations.

 Minor comments:

 I could not find the method for generating 10X genomics read data in the sequencing section. It will be good if the authors can add a short description of the method.

 For genome assembly, curation and evaluation I would request the authors to add maybe in brief, some of the important parameters that were used for each program.

 Also I will request the authors to publicly share the scripts ( maybe though github page) necessary to perform the analyses.

Are sufficient details of methods and materials provided to allow replication by others?

Yes

Is the rationale for creating the dataset(s) clearly described?

Yes

Are the datasets clearly presented in a useable and accessible format?

Yes

Are the protocols appropriate and is the work technically sound?

Yes

Reviewer Expertise:

NGS, Comparative Genomics, Population and Phylogenomics, Evolution, Biodiversity genomics.

I confirm that I have read this submission and believe that I have an appropriate level of expertise to confirm that it is of an acceptable scientific standard.

# Reviewer report: Reviewer response for version 1

**DOI:** 10.21956/wellcomeopenres.22030.r81217

**Published:** 2024-05-14

**Authors:** Xiuguang Mao

**Affiliations:** 1 East China Normal University, Shanghai, China

Ruedi et al. present a high-quality chromosome-scale assembly for *Pipistrellus pygmaeus*. The ends of most of the chromosomal-scale scaffolds have telomere sequences, supporting the completeness of this assembly. In addition, the total number of chromosomal-level scaffolds corresponds to the haploid chromosome number of this species (2n=44). This genome provides valuable resource to study the genomic divergence of this species from other *Pipistrellus *( *P. pipistrellus*, *P. kuhlii* and *P. nathusii*) which also have high-quality assemblies.

 One minor comment or suggestion:

 Can author explain a little bit for “The sex chromosomes were assigned by coverage statistics” in methods? or use a figure to show the coverage difference of reads mapping for X and Y chromosomes compared to autosomes?

Are sufficient details of methods and materials provided to allow replication by others?

Yes

Is the rationale for creating the dataset(s) clearly described?

Yes

Are the datasets clearly presented in a useable and accessible format?

Yes

Are the protocols appropriate and is the work technically sound?

Yes

Reviewer Expertise:

Comparative genomics, genome assembly, speciation, chromosome evolution, bats

I confirm that I have read this submission and believe that I have an appropriate level of expertise to confirm that it is of an acceptable scientific standard.

# Reviewer report: Reviewer response for version 1

**DOI:** 10.21956/wellcomeopenres.22030.r66165

**Published:** 2023-08-30

**Authors:** Matthew Christmas

**Affiliations:** 1 Department of Medical Biochemistry and Microbiology, Science for Life Laboratory, Uppsala University, Uppsala, Uppsala County, Sweden

Ruedi *et al. *provide a description of the chromosome-level assembly of the *Pipstrellus pygmaeus* genome, including the assembly of PacBio HiFi reads, assembly polishing, scaffolding, and mitogenome assembly. The assembly pipeline is appropriate and all assembly steps are clearly described. This genome provides an excellent resource as a basis for studying the evolution of this species and its divergence from other chiropterans, including the closely related *Pipistrellus pipistrellus.*

 One minor comment:

- "The estimated Quality Value (QV) of the final assembly is 61" - it would be good to provide context for this, does a score of 61 suggest high quality?

Are sufficient details of methods and materials provided to allow replication by others?

Yes

Is the rationale for creating the dataset(s) clearly described?

Yes

Are the datasets clearly presented in a useable and accessible format?

Yes

Are the protocols appropriate and is the work technically sound?

Yes

Reviewer Expertise:

Evolutionary genomics, population genomics, comparative genomics, genome assembly

I confirm that I have read this submission and believe that I have an appropriate level of expertise to confirm that it is of an acceptable scientific standard.
